# Assessment of the antidiabetic potential of extract and novel phytoniosomes formulation of *Tradescantia pallida* leaves in the alloxan‐induced diabetic mouse model

**DOI:** 10.1096/fj.202201395RR

**Published:** 2023-03-01

**Authors:** Fariha Imtiaz, Muhammad Islam, Hamid Saeed, Abrar Ahmed, Hassaan Anwer Rathore

**Affiliations:** ^1^ Section of Pharmaceutical Chemistry, Punjab University College of Pharmacy, Allama Iqbal Campus University of the Punjab Lahore Pakistan; ^2^ Section of Pharmaceutics, Punjab University College of Pharmacy, Allama Iqbal Campus University of the Punjab Lahore Pakistan; ^3^ Section of Pharmacognosy, Punjab University College of Pharmacy, Allama Iqbal Campus University of the Punjab Lahore Pakistan; ^4^ Department of Pharmaceutical Sciences, College of Pharmacy QU Health, Qatar University Doha Qatar

**Keywords:** bioactive compounds, diabetes, molecular docking, phytochemicals, phytoniosomes, *Tradescantia pallida*

## Abstract

Diabetes inflicts health and economic burdens on communities and the present antidiabetic therapies have several drawbacks. *Tradescantia pallida* leaves have been used as a food colorant and food preservative; however, to our knowledge antidiabetic potential of the leaves of *T. pallida* has not been explored yet. The current study aimed to investigate the antidiabetic potential of *T. pallida* leaves extract and its comparison with the novel nisosome formulation of the extract. The leaves extract and phytoniosomes of *T. pallida* in doses of 15, 25 and 50 mg/kg were used to assess the oral glucose loaded, and alloxan‐induced diabetic mice models. The biological parameters evaluated were; change in body weight, blood biochemistry, relative organ to body weight ratio and histopathology of the liver, pancreas and kidney. Results revealed that the extract 50 mg/kg and phytoniosomes 25 and 50 mg/kg remarkably reduced the blood glucose level in all hyperglycemic mice by possibly inhibiting α‐amylase and α‐glucosidase production. Body weight and blood biochemical parameters were considerably improved in phytoniosomes 50 mg/kg treated group. The relative body weight was similar to those of healthy mice in extract 50 mg/kg, phytoniosomes 25 mg/kg, and phytoniosomes 50 mg/kg treated groups. Histopathology showed the regeneration of cells in the CHN50 treated group. Hyphenated chromatographic analysis revealed potent metabolites, which confirmed the antidiabetic potential of the extract by inhibiting α‐amylase and α‐glucosidase using in silico analysis. The present data suggested that phytoniosomes have shown better antidiabetic potential than crude extract of these leaves.

## Graphical abstract

The graphical abstract depicts the potential of novel phytoniosomes formulation in comparison with the parent extract. The antidiabetic potential of the extract and phytoniosomes was checked by monitoring blood glucose level for 28 days by employing mice models. After 28 days, blood biochemical test and histopathological analysis revealed remarkable antidiabetic potential of phytoniosomes when compared with standard and extract. The mechanism of action and drug‐likeness of the putative compounds responsible for antidiabetic activity was validated using in silico models.

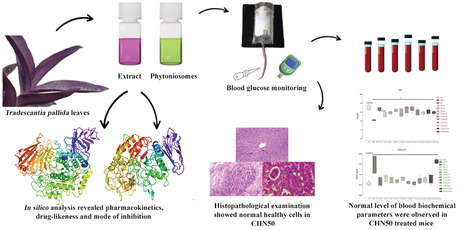

**Abbreviations**
ALB: albumin
ALP: alkaline phosphatase
ALT: alanine transaminase
AST: aspartate aminotransferase
BGL: blood glucose level
CHN: phytoniosomes of *Tradescantia pallida*
CREAT: creatinine
GC–MS: gas chromatography mass spectroscopy
HDL‐C: high‐density lipoproteins
LDL‐C: low‐density lipoproteins
SU: serum urea
TBIL: total bilirubin
TC: total cholesterol
TG: total glycerides
TP: total protein
TP: *Tradescantia pallida*
UA: uric acid
VLDL: very low‐density lipoproteins

## 1 INTRODUCTION

In a survey in 2019, 463 million people between 20 and 79 years of age died of diabetes, and this number is expected to rise to 578 million in 2030 and 700 million by 2045.^1^ Likewise, worldwide expenditures on diabetes are set to be around 760 billion USD, which will increase to 825 billion USD by 2030 and up to 845 billion USD by the time we reach 2045. Pakistan has been ranked number three followed by China and India in the prevalence of diabetes, with 33 million people living with this chronic disease.^2^ Management of diabetes encompasses a healthy lifestyle with weight loss, exercise, oral and injectable hypoglycemic agents, however, none of them could cure the underlying cause of the disease and the illness itself.^3^, ^4^ Although many antidiabetic drugs of synthetic and natural origin are already in the market, diabetes with its complications is still one of the top major health issues worldwide.^5^ The therapeutic potential of the current antidiabetic drugs is marred by several limitations, such as poor efficacy, unaffordability and innumerable side effects including hepatic dysfunction, weight gain, weak eyesight, neuropathy and gastrointestinal discomfort.^6^, ^7^

Taking into account the clinical incapacitation of the conventional medicines, herbal drugs with claimed antidiabetic potential can be used as an alternative therapy to treat diabetes, due to easy accessibility, cost‐efficiency and sweeping cultural adaptability with fewer side effects.^8^ Worldwide, more than 1200 plant species have been utilized by different ethnic communities for their presumed antidiabetic activity as traditional medicine. However, hardly half of these species have been validated for their proclaimed scientific use.^9^, ^10^ One such plant genus Tradescantia claims to have the antidiabetic potential.^11^ *Tradescantia zebrina* leaves are used for the preparation of infusions to treat diabetes by Mexicans.^12^ While the species *Tradescantia spathacea* is consumed in Puerto Rico for lowering blood glucose levels,^13^ the large gap of knowledge in the ethnobotanical evaluation of *Tradescantia pallida* has probed us to investigate its antidiabetic potential. *T. pallida* grows annually in subtropical and tropical regions of Asian countries including Pakistan. *T. pallida* have various reported pharmacological activities; mainly, its use for sore eyes, as an antiinflammatory agent and as an antioxidant. *T. pallida* contains flavonoids, alkaloids, tannins and terpenoids, which may slow down and reduce the absorption of the whole extract.^14^ Therefore, a novel drug delivery system is an urgent need of the hour that can enhance the bioavailability of herbal drugs, reduce dose size and frequency of administration.

Drugs with poor bioavailability have been in the investigation for finding a novel drug delivery system, for the last few decades, and have been successful in introducing nanoparticles, micro emulsions, liposomes and niosomes.^15^, ^16^, ^17^, ^18^, ^19^, ^20^, ^21^, ^22^, ^23^, ^24^ Out of all new delivery systems, niosomes came out to be promising candidates resulting in enhanced solubility, better bioavailability in overcoming the side effects linked with the utilization of whole crude plant extracts. Niosomes are spherical, non‐ionic surfactants forming bi‐layer membranes having good chemical and storage stability with cost‐effective preparation methods.^25^, ^26^ Successful experiments have already been carried out showing promising therapeutic effects of plant‐based niosomes including *Ginkgo biloba*, *Curcuma longa* Linn, *Silybum marianum*, and *Gymnema sylvestre*.^26^, ^27^, ^28^

The current study aimed to investigate in vivo antidiabetic potential of *T. pallida* leaves extract and *T. pallida* extract‐loaded phytoniosomes using the mouse model and validating it through histopathology of the organs. The in vivo antidiabetic effect of the optimum niosomal *T. pallida* leave formulation has been compared with *T. pallida* chloroform extract to conclude the results.

## 2 MATERIALS AND METHODS

### 2.1 Materials

Chloroform of analytical grade (Emsure, Merck Millipore, KGaA, USA) was used for solid–liquid extraction of *T. pallida* leaves; Span 60 (Sigma‐Aldrich Chemire GmbH, Germany) was used as a non‐ionic surfactant; cholesterol (Sigma‐Aldrich Chemire GmbH, Germany) acted as a membrane stabilizing agent; distilled water (prepared using Merit water still assembly from Stuart, Cole‐Parmer, UK) and acarbose (Sigma‐Aldrich Chemire GmbH, Germany) were used as standard reference materials; anhydrous glucose (Calbiochem, Merck Millipore KGaA, Germany) was used to glycate with hemoglobin; assay kits for the analysis of each blood biochemical parameter were purchased from Sigma‐Aldrich Chemire GmbH, Germany; and Alloxan monohydrate (Sigma‐Aldrich Chemire GmbH, Germany) was used to induce diabetes in mice.

### 2.2 Preparation of plant extract

*T. pallida* (common name purple‐heart; English name purple queen) leaves were purchased from local plant nurseries of Lahore, Pakistan. Taxonomist from Government College University (GCU), Lahore, Pakistan identified and authenticated the plant and issued a voucher number GC. Herb. Bot. 3627. Shade dried leaves were pulverized and solid–liquid hot extraction was carried out using soxhlet extraction assembly. Precisely, 250 g of powdered leaves were packed in a thimble and were extracted against 4.5 L of chloroform. After extraction, the extract was dried using a rotary evaporator (Heidolph Rotary Evaporator, Laborota 4002, Merck, KGaA, Germany) and stored at 4°C.^29^

### 2.3 Molecular spectroscopic analysis of the extract

#### 2.3.1 UV–Vis analysis

Chloroform extract of leaves of *T. pallida* was diluted to 1:10 with methanol. The sample was scanned from 200 to 800 nm using UVprobe software (UV–Vis 2550, Shimadzu Corporation, Japan).

#### 2.3.2 FTIR analysis

The infrared spectrum of the extract was recorded in the wave number ranging from 4000 to 650 cm^−1^ at ambient temperature using (Agilent MicroLab software, Cary 630 FTIR Spectrometer, Agilent, USA).

### 2.4 Proximate analysis and quantification of secondary metabolites

#### 2.4.1 Proximate analysis

Moisture content was calculated by drying 2 g powdered material at 105°C with the interval of 30 min until constant weight achieved.^30^ The percentage of total ash was calculated by incinerating 2 g powdered sample in a muffle furnace at 675 ± 25°C until carbon free.^30^ The total protein content was estimated by following the method of Lowry.^31^ Total lipids were estimated by extracting the powder with n‐hexane and weighing the dry extract.^32^ Total carbohydrates were calculated using formula,^33^

$$\begin{array}{c} \text{Percentage total carbohydrates}=100-\left(\text{total}\;\mathrm{ash}\right.\\ \;\left.+,\text{total moisture},+,\text{total},\;,\mathrm{fat},+,\text{total proteins}\right). \end{array}$$

#### 2.4.2 Total polyphenolic content

The total polyphenolic content of the chloroform extract was calculated using Gallic acid as standard (mg GAE/g). Standard stock solution (1 mg/mL) of gallic acid and sample extracts were prepared in methanol. The concentration series of gallic acid solution was prepared and 200 μL was taken out from each sample. 200 μL Folin–Ciocalteu reagent and 1 mL 15% sodium carbonate was added and volume was made up to 3 mL with methanol. All mixtures were incubated for 90 min at room temperature and absorbance was measured at 760 nm. A standard calibration curve was drawn to estimate total polyphenolic contents.^34^

#### 2.4.3 Total flavonoid content

Quercetin was used as a standard to quantify flavonoids in chloroform extract. Standard and extract were prepared in methanol (1 mg/mL). The concentration series from the quercetin stock solution was prepared. 200 μL was taken from quercetin dilutions and chloroform extract and volume was made up to 1 mL with methanol. To each solution, 100 μL of 10% aluminum nitrate and 100 μL of 1 M potassium acetate were added and the final volume was made up to 5 mL with distilled water. After incubating at room temperature for 45 min, absorbance was measured at 415 nm. The concentration of total flavonoid was determined as mg equivalents of quercetin per g.^35^

#### 2.4.4 Estimation of total polysaccharides

Eighty percent (80%) hot ethanol (7 mL) was added to a 200 mg extract and centrifuged at 2700 rpm for 10 min. Supernatant was collected and procedure was repeated until supernatant gave no green color with anthrone reagent. Collected residue was dried and extracted with a mixture of 10 mL distilled water and 25% HCl (1:1 v/v) for 20 min at 0°C and centrifuged at 2700 rpm for 10 min. 100 μL of aliquots were taken and volume was made up to 1 mL with distilled water followed by the addition of 4 mL anthrone reagent. Mixtures were heated in a water bath for 10 min and intensity of green color was measured at 600 nm using UV–Visible spectrophotometer against glucose as standard.^36^

#### 2.4.5 Estimation of total glycosaponins

Briefly, 1 g of each extract was dissolved in 50 mL methanol and refluxed for 30 min using reflux assembly. Solutions were then filtered and dried to 10 mL using a rotary evaporator. A tarred beaker with 50 mL acetone was taken and extracted solutions were added drop wise to get precipitates. These precipitates were dried in the oven at 100°C and the quantity of glycosaponins were calculated by the following formula^36^:

$$\text{Total glycosaponins}\%=\frac{\text{weight of precipitate}}{\text{weight of sample}}\times 100.$$

### 2.5 Synthesis of phytoniosomes

The ultra probe sonication technique was used to synthesize phytoniosomes.^37^, ^38^ The dried chloroform extract was dissolved in double‐distilled water (1: 10 w/v %) for 2 h, 37°C, and at 2000 rpm. Briefly, 100 μL from prepared stock solution was mixed in 12 mL of double‐distilled water using a magnetic stirrer. Cholesterol and Span 60 were admixed and were subjected to probe sonication (Q125 sonicator, Thomas Scientific, USA) at 57°C/5 min in a pulsatile mode (10‐s pause), at an amplitude of 30%. Prepared extract‐loaded phytoniosomes were collected and stored at 4°C until further use.^39^

### 2.6 Characterization of phytoniosomes

#### 2.6.1 Particle size analysis

The particle size of the phytoniosomes was determined using photon correlation spectroscopy (Malvern Instruments, USA). Briefly, 20 μL of sample was dissolved in 15 mL double distilled water and size was measured.

#### 2.6.2 Entrapment efficiency

The niosomal formulation was centrifuged for an hour at 14 000 rpm and 4°C to determine the entrapment efficiency (Centrifuge 2–16 KC, Sigma). The supernatant was collected, and pellet underwent two washes with distilled water. In pooled supernatants, extract entrapment measurement was calculated using the following formula^40^:

$$\%\text{Entrapement efficiency}=\left[\left(\mathrm{Qe}-\mathrm{Qp}\right)/\mathrm{Qe}\right]\times 100,$$

where *Qe* is the extract's amount used for phytoniosomes synthesis and *Qp* is the extract's amount present in the supernatant.

#### 2.6.3 Morphology

The shape of the phytoniosomes was determined using TEM (Transmission electron microscopy). Using a 2% uranyl acetate solution, samples were negatively stained. The control niosome formulation was further diluted to prevent aggregation and to identify individual particles. Before measurements, samples were produced using carbon‐coated copper mesh and air dried at room temperature.

#### 2.6.4 In vitro extract release study

Membrane dialysis method was used to liberate the extract from niosome formulations and the pure extract. An overnight‐soaked dialysis membrane was treated with 10 mg of the formulation dispersed in 5 mL of PBS pH 7.4. The membrane pack was then inserted into 100 mL of PBS and mixed at 100 rpm while the mixture was kept at 37°C. At predefined intervals of time (0, 1, 2, 3, 4, 6, 8, 10 and 12 h), 3 mL samples were collected. The same amount of new PBS was used to replace the withdrawn sample. A UV–Vis spectrophotometer method (UV‐2550, Agilent Technologies) was used to measure the extract release at a wavelength of 279 nm.^41^

### 2.7 Experimental animals

Mouse provides the most accessible mammalian model by sharing similar organ systems, physiology and genome patterns with humans.^42^ Despite the availability of various in vitro models, the mouse model is still an effective one to understand the complex multi‐systemic interactions in diabetes.^43^ Keeping this in view, the current project was designed to include pre‐clinical data that could be extrapolated for human benefit in better control of diabetes mellitus. Male and female Swiss albino mice weighing (30–32 g) in the age range from 10 to 12 weeks were purchased from the University of Veterinary and Animal Sciences, Lahore, Pakistan. Mice were acclimatized in SPF‐level lab for 7 days at 24 ± 2°C, 12 h light/dark cycle.^44^ A total of 91 mice were procured at different stages of the study and randomly allocated. Guide for the Care and Use of Laboratory Animals instructions were followed in all the procedures carried out in the experiments.^45^ ARRIVE 2.0 guidelines were followed for animal research reporting standards and power analysis method was chosen for sample size determination. Punjab University Institutional Ethics Review Board approved the use and sacrifice of animals (ethical approval number 192/DFEMS).Grimace mouse scale^46^ was used to determine humane endpoints. If the scale indicated “moderately present” signs of pain then oral pediatric ibuprofen 20 mg/kg, ad libitum, in drinking water was to be given and for scale “obviously present” animals were to be euthanized with anesthesia over dose using pentobarbital sodium 200 mg/kg intraperitoneally.

### 2.8 Acute toxicity study

Organization for Economic Cooperation and Development (OECD) No 425 Guideline was followed to carry out an acute toxicity test of extract and phytoniosomes of leaves of *T. pallida*, based on the principle of the limit test. On the first day of the experiment, 1000 and 2000 mg/kg extract and phytoniosome (equivalent to the weight of parent extract) were given by oral gavage to two female Swiss albino mice who were fasted for 4 h (water ad libitum). The mice were housed separately and their physical and behavioral changes were strictly observed for 4 h, emphasizing changes in the fur, lacrimation, reduction in feeding activity, frequency of urination, paw licking, itching, reduced motor activity, weight loss, diarrhea, and paralysis. Mice were kept under observation for the next 24 h. Based on the expressions collected on the first day, the other 4 mice (divided in each group) were fasted for 4 h and were given similar doses of 1000 and 2000 mg/kg body weight orally in a single dose. The animals were kept under observation for 28 days for any sign of gross behavioral change and toxicity, mentioned above. Surviving mice were weighed and then humanely sacrificed on day 28 to collect blood, liver, kidney and pancreas for histopathology and organ to bodyweight ratio calculations were recorded.^47^

### 2.9 Experimental grouping of mice and dosing

In the following procedures, male mice were used in all experimental groups due to the less sensitivity of female mice to insulin.^48^ Glucose‐loaded tolerance and long term effect in alloxan‐induced diabetic mice models were put on trial to investigate the antidiabetic potential of leaves extract and phytoniosomes of *T. pallida*. Mice were divided into 8 groups for glucose‐loaded tolerance including 5 mice in each group. Normal control received distilled water (NC), positive control received acarbose 50 mg/kg (PC), 15 mg/kg extract (TP15), 25 mg/kg extract (TP25), 50 mg/kg extract (TP50), 15 mg/kg (equivalent to extract) phytoniosomes (CHN15), 25 mg/kg (equivalent to extract) phytoniosomes (CHN25) and 50 mg/kg (equivalent to extract) phytoniosomes (CHN50). For multiple dose‐treated groups, mice were divided into 9 groups with 5 mice in each group. Normal control received distilled water (NC), negative controls, alloxan‐induced diabetic control, received distilled water (NEC), positive control diabetic mice, received standard drug acarbose 50 mg/kg (PC). In the test group, alloxan‐induced diabetic mice received 15 mg/kg extract (TP15), 25 mg/kg extract (TP25), 50 mg/kg extract (TP50), 15 mg/kg (equivalent to extract) phytoniosomes (CHN15), 25 mg/kg phytoniosomes (CHN25) and 50 mg/kg (equivalent to extract) phytoniosomes (CHN50). The groups were similar as mentioned above with an addition of a negative control group (NEC) that only received distilled water.

### 2.10 Development of alloxan‐induced diabetes mellitus mouse model

Diabetes was induced using alloxan monohydrate. Alloxan monohydrate was prepared in cold normal saline 0.9% (pH 5.5). A 150 mg/kg dose of the freshly prepared alloxan monohydrate solution was injected intraperitoneally to 18 h fasted mice. Food and water were provided after 30 min of injection. 5% of glucose solution (1 mL/kg) was given to mice after 6 h of injection for the next 24 h to prevent sudden death from hypoglycemic shock. After 72 h of alloxan injection, diabetes screening was done using a glucometer (ACCU‐Chek Performa blood glucose meter, Roche, Switzerland). Overnight fasting mice with blood glucose levels >250 mg/dL were considered diabetic and were randomly divided into different experimental groups.

### 2.11 Oral glucose tolerance test (OGTT) in normoglycemic mice on extract and phytoniosomes of *T. pallida* leaves

Overnight fasted normoglycemic mice were randomly divided into 8 groups. After half an hour of administration of extract and phytoniosomes of *T. pallida* leaves, 2000 mg/kg glucose solution was orally given to each mouse. Blood glucose level was measured at 0, 30, 60, 90, and 120 min of post glucose oral gavage.

### 2.12 Hypoglycemic effect of *T. pallida* leaves crude extract and phytoniosomes in alloxan‐induced diabetic mice

Overnight fasting mice were divided into 9 groups with 5 mice in each experimental group and were treated once daily for 28 days. Blood glucose level was measured at day 0, 14, and 28 along with mean BGL of each group was recorded with standard deviation.

### 2.13 Bodyweight and blood biochemical measurements

In repeated daily dose treatment, change in body weight of mice was recorded before induction of diabetes (pre‐treatment), at day 0, 14th day (during treatment), and 28th day (post‐treatment).

After the completion of in vivo activity mice were sacrificed by cervical dislocation and blood was withdrawn via cardiac puncture for the analysis of biochemical parameters. The serum was tested for TC, TG, HDL‐C, LDL‐C, VLDL, CREAT, SU, UA, TBIL, ALT, ALP, AST, TP, and ALB using assay kits.

### 2.14 Organ to body weight ratio

After sacrificing, mice were dissected to separate liver, kidney, liver, and pancreas. Organs were weighed in grams using a weighing balance (BL 2005, Setra, USA) and their relative weights were calculated using the following formula^49^:

$$\text{Relative weight}\%=\frac{\text{organ weight of mouse}}{\text{body weight of mouse}}\times 100.$$

### 2.15 Histopathology of mice organs

The liver, kidney and pancreas were carefully removed from the mice's bodies and were fixed in 10% formalin for 24 h. Organs were then embedded in paraffin wax and thin slices of 5 μm were carefully cut and stained using hematoxylin and eosin (H&E). These thin, stained sections of each organ were converted into permanent slides, and histological changes were observed using an inverted light microscope (Labomed TCM 400, USA).

### 2.16 Hyphenated chromatographic analysis

Bioactive metabolites were detected using hyphenated chromatography. The system consisted of GC–MS (GC system 7890A, Agilent Technologies, USA, and MS 5975C Agilent Technlogies, USA). A capillary column (DB‐35 MS) with length 15 m, internal diameter 0.25 nm, and 0.25 μM film thickness was used for the separation of metabolites. Helium was used as a carrier gas at a flow rate of 1 mL/min. Then, 1 μL sample was injected at splitless inlet mode with column pressure 0.77 psi. Starting oven temperature was 100°C for 5 min, which increased to 200°C at 1°C/min rate and kept for 10 min. Finally, the temperature was raised to 320°C for 30 min and held for 20 min. The fragment spectra of each metabolite were compared to the NIST20 Database with a minimum quality factor 80.

### 2.17 In silico evaluation

Schrödinger suite (version 13.2, LLC, 2022.2, New York, NY, USA) was used for in silico analysis of GC–MS identified compounds.^50^, ^51^

#### 2.17.1 ADME profile

Absorption, distribution, metabolism and excretion (ADME) characteristics of the selected compounds were analyzed using the QikProp® module (Schrödinger, version 13.2, LLC, 2022.2, New York, NY, USA).

#### 2.17.2 Molecular docking and Prime MM‐GBSA energy calculations

Human α‐amylase (PDB: 4GQR) and α‐glucosidase (PDB: 5NN5) were retrieved from protein data bank. Protein Preparation Wizard was used to refine the protein structures. The structures of the compounds were prepared using 2D sketch mode in Maestro. The compounds were prepared using the LigPrep module and energy was minimized by optimized potentials for liquid simulations (OPLS 2005) force field. Grid box was generated in 30 × 30 × 30 Å dimension using the Glide module. Extra precision (XP) mode was used for ligand docking calculations. The first three compounds with the highest docking scores were selected for Prime MM‐GBSA calculations. Prime MM‐GBSA energy calculations were performed by applying force field (OPLS 2005) and generalized‐Born/surface area continuum solvent model. The 3D ligand‐receptor structures were prepared using Pymol (v.2.5.2, Schrödinger, New York, NY, USA).

### 2.18 Statistical analysis

All the data were presented as mean ± standard deviation. Analysis was carried out using one‐way ANOVA with Tukey's post hoc test and two‐way analysis of variance followed by post hoc Sidak test to compare groups using GraphPad prism (version 8.0.1, California, USA). *p* values <.05 were considered statistically significant.

## 3 RESULTS

### 3.1 Molecular spectroscopic analysis of extract

**FIGURE 1:**
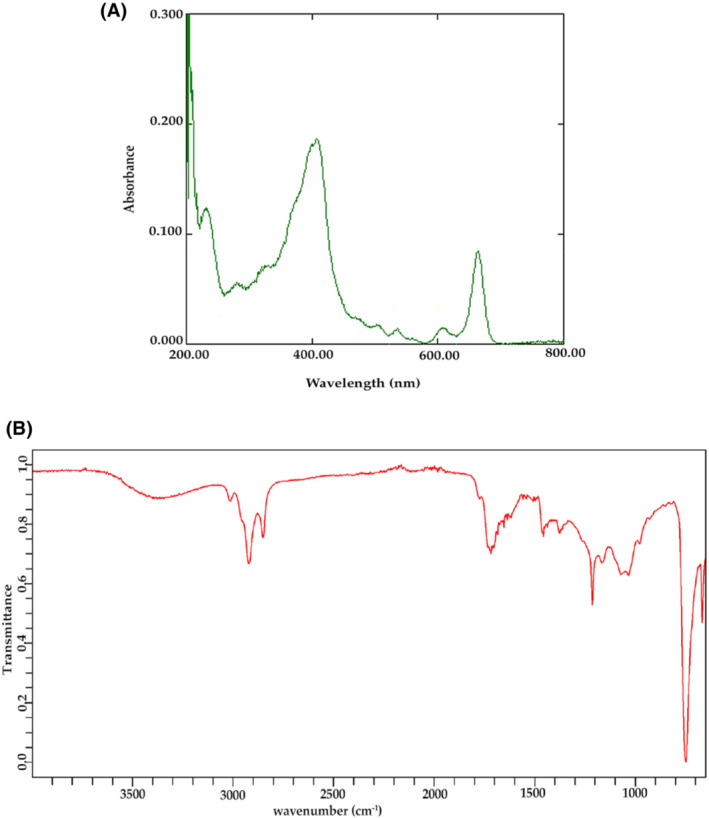
Molecular spectroscopic analysis of chloroform extract of leaves of *Tradescantia pallida* (A) UV–Visible spectra, (B) FTIR spectra.

Qualitative analysis to identify secondary metabolites was done using UV–Vis and FTIR spectroscopy. The UV profile of chloroform extract showed peaks at 235 and 279 nm. While, in the visible range, the peaks observed were at 410, 540, 612, and 662 nm (Figure 1A).

FTIR spectroscopic analysis of chloroform extract detected functional groups in seven different frequency regions. The characteristic peak observed at 3022.01 cm^−1^ corresponds to O–H, C–O, and N–H stretching vibrations. The peaks at 2919.78 and 2849.48 cm^−1^ showed C–H stretching vibration. The C=O and N–H bonds were observed at peak 1799.39 cm^−1^. While the peak at 1399.35 cm^−1^ showed O–H, COO– and C–H, NH_2_ and C–N bonds. While, the peaks at 749.12 and 656.30 cm^−1^ could be due to the C–H bending vibrations (Figure 1B).

### 3.2 Proximate analysis and secondary metabolites

The results revealed the presence of high content of polyphenols and flavonoids in the chloroform extract of leaves of *T. pallida* (Table 1).

**TABLE 1:** Proximate analysis and secondary metabolites in the chloroform extract of leaves of *Tradescantia pallida*.

No.	Proximate analysis	Chloroform extract
		Mean ± SD
1	Moisture content (%)	9.45 ± 0.21
2	Total ash (%)	15.4 ± 0.22
3	Total lipids (w/w)	5.44 ± 0.07
4	Total proteins (w/w)	9.27 ± 0.40
5	Total carbohydrates (w/w)	64.08 ± 0.19

	Secondary metabolites	Chloroform extract
		Mean ± SD
6	Total polyphenols (mg GAE/g)	353.67 ± 0.13
7	Total flavonoids (mg Que/g)	240.83 ± 0.29
8	Total polysaccharides (mg Glu/g)	205.20 ± 0.05
9	Total glycosaponins (%)	11.66 ± 0.28

*Note*: The values are presented as mean ± standard deviation.

### 3.3 Characterization of phytoniosomes

Small, homogenous particle size (<350 nm) and good entrapment efficiency (>80%) were selected Critical Quality Attributes (CQA) for phytoniosomal formulation. The results revealed that the particle size and PDI value met the CQA standards (Table 2). Niosomes that were empty had an average particle size of 214 nm and a PDI score of 0.213. The particle size was reduced to 160 nm when drug‐loaded niosomes were created using the same formula as the empty niosomes. The entrapment efficiency achieved was 89.12%.

**TABLE 2:** Particle size, PDI value and entrapment efficiency of novel phytoniosomes formulation.

Formulation	Size (nm)	PDI	% Entrapment efficiency
Control formulation	214.15 ± 0.12	0.213 ± 0.013	–
Phytoniosomes formulation	160.11 ± 0.18	0.134 ± 0.010	89.12 ± 0.91

**FIGURE 2:**
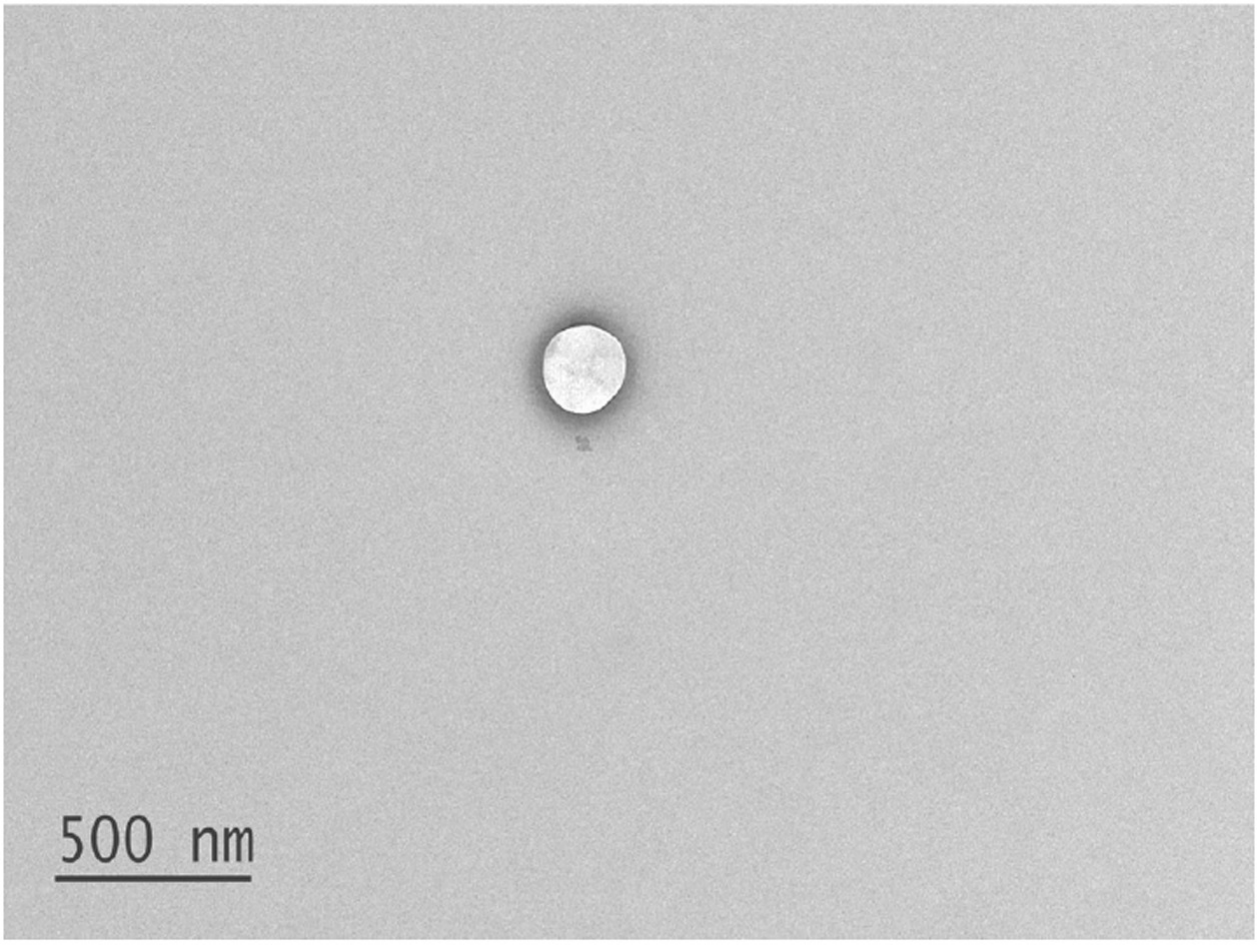
TEM image of control niosome depicting spherical morphology.

The morphology of phytoniosomes was revealed by TEM. The primary objective was to determine the morphology and form of niosomes. The formulation diluted prior to TEM examination to make sure that individual niosome was examined. The typical size and form of a control niosome is shown in Figure 2. Niosomes had a sphere shape, and the size was in good agreement with particle size data that was previously mentioned in Table 2.

**FIGURE 3:**
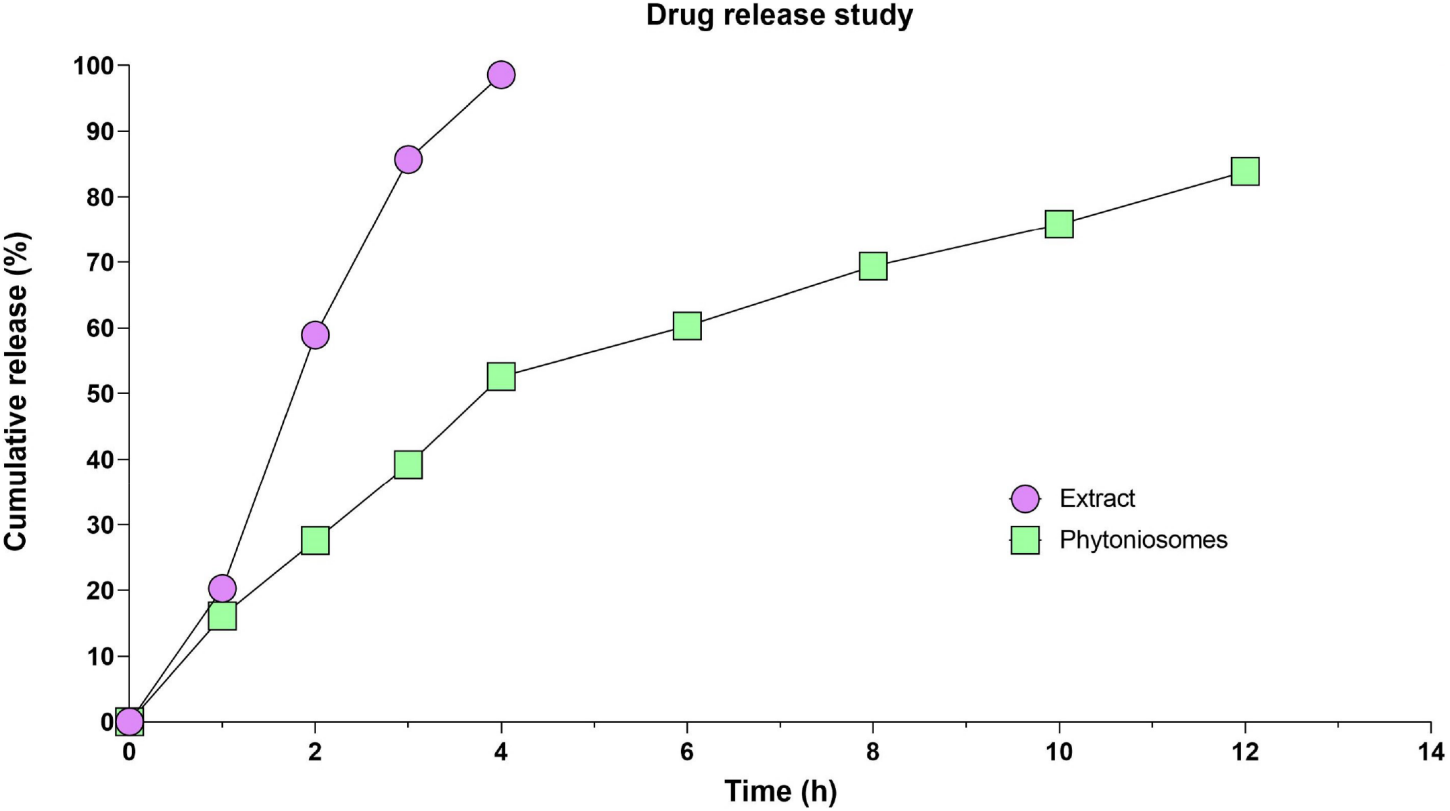
Drug release profile of extract and phytoniosomes of leaves of *Tradescantia pallida*.

The biphasic release profile was observed in drug release study. The formulation showed small burst release in first 4 h followed by a slow and controlled release (Figure 3). However, on the other side, the extract showed a very fast release profile, releasing the entire drug within 4 h.

### 3.4 Acute toxicity test

The test showed no mortality and any signs of visible toxicity including any physical, neurological, or behavioral changes in the first 24 h and then for 28 days. Concluded from the mortality rate, the dose of 2000 mg/kg of body weight of the crude extract and phytoniosomes of *T. pallida* leaves can be considered safe.

### 3.5 Oral glucose loaded tolerance in normoglycemic mice

After administration of the test sample, the blood glucose levels of mice in all groups at 0 min were in the normal range (Table 3). After 30 min, blood glucose levels in all groups were significantly high regardless of the treatment. Intergroup analysis manifested a significant decrease in blood glucose level at 60 min that persisted for another 60 min. When BGL was compared at 30 min with the rest of the groups, all TP and CHN groups showed statistically outstanding results (*p* < .001).

**TABLE 3:** Oral glucose tolerance activity in normoglycemic mice.

Blood glucose level (mg/dL)					
Groups	0 h	30 min	60 min	90 min	120 min
	Mean ± SD				
NC	89.66 ± 0.57	183.66 ± 4.72	195.00 ± 2.00	174.00 ± 6.24	116.00 ± 13.89
PC	89.33 ± 0.57	193.33 ± 6.65	123.66 ± 14.01	127.33 ± 3.78	98.00 ± 7.21
TP15	94.33 ± 5.03	201.33 ± 6.80	176.00 ± 7.21	141.66 ± 3.05	109.66 ± 0.57
TP25	82.33 ± 3.21	189.33 ± 1.15	144.66 ± 6.02	113.00 ± 5.29	82.00 ± 3.60
TP50	92.66 ± 3.78	202.33 ± 2.30	161.33 ± 6.80	81.00 ± 6.55^a^ ^,^ *	76.33 ± 5.03^a^ ^,^ **
CHN15	89.00 ± 5.56	193.00 ± 2.08	109.33 ± 2.08^b^ ^,^ ^c^ ^,^ ^d^ ^,^ *	108.66 ± 2.51^b^ ^,^ *	110.66 ± 2.08
CHN25	93.33 ± 3.51	184.66 ± 6.80	73.66 ± 5.50^b^ ^,^ ^c^ ^,^ ^d^ ^,^ *	72.66 ± 5.50^b^ ^,^ ^c^ ^,^ *	74.66 ± 5.50^b^ ^,^ *
CHN50	106.00 ± 2.64	208.33 ± 1.52	74.33 ± 7.63^b^ ^,^ ^c^ ^,^ ^d^ ^,^ *	70.66 ± 10.11^b^ ^,^ ^c^ ^,^ *	67.66 ± 12.05^b^ ^,^ *

*Note*: Values are presented in mean ± SD, one way ANOVA post hoc Tukey's test.

Abbreviations: CHN15, niosome group 15 mg/kg; CHN25, niosome group 25 mg/kg; CHN50, niosome group 50 mg/kg; NC, normal control; PC, positive contol; TP15, extract group 15 mg/kg; TP25, extract group 25 mg/kg; TP50, extract group 50 mg/kg.

^a^ Compared with PC.

^b^ Compared with TP15.

^c^ Compared with TP25.

^d^ Compared with TP50.

* *p* < .001

** *p* < .05.

### 3.6 Hypoglycemic effect in alloxan‐induced diabetic mice

Analysis between the test groups revealed a considerable decline in blood glucose levels at days 14 and 28 when compared with their respective baseline values (Table 4).

**TABLE 4:** Hypoglycemic effect in alloxan‐induced diabetic mice.

Blood glucose level (mg/dL)					
Groups	Baseline	Day 14	Day 28	Reduction Day 14	Reduction Day 28
	Mean ± SD, *p*‐value			%	
NC	90.80 ± 4.60	88.80 ± 5.31	87.40 ± 6.26	2.2	3.74
NEC	396.60 ± 4.09	391.40 ± 7.63	397.60 ± 4.77	1.31	−0.25
PC	372.00 ± 5.04	166.00 ± 20.65	136.40 ± 13.06	55.37	63.44
TP15	367.00 ± 39.20	336.00 ± 17.56	327.60 ± 14.2	8.44	10.73
TP25	393.80 ± 15.51	280.00 ± 38.88	247.00 ± 21.45	28.89	36.99
TP50	383.60 ± 8.64	240.20 ± 16.39	198.00 ± 12.94	37.38	48.38
CHN15	388.20 ± 11.03	353.00 ± 19.64	336.60 ± 14.77	9.06	13.44
CHN25	399.40 ± 9.01	253.60 ± 14.75^b^ ^,^ *	153.40 ± 25.40^b^ ^,^ ^c^ ^,^ ^d^ ^,^ *	36.5	61.59
CHN50	389.80 ± 14.73	139.60 ± 13.37^b^ ^,^ ^c^ ^,^ ^d^ ^,^ *	81.40 ± 8.20^a^ ^,^ ^b^ ^,^ ^c^ ^,^ ^d^ ^,^ *	64.18	79.11

*Note*: Values are presented in mean ± SD, two‐way ANOVA post hoc Sidak's test.

Abbreviations: CHN15, niosome group 15 mg/kg; CHN25, niosome group 25 mg/kg; CHN50, niosome group 50 mg/kg; NC, normal control; PC, positive contol; TP15, extract group 15 mg/kg; TP25, extract group 25 mg/kg; TP50, extract group 50 mg/kg.

^a^ Compared with PC.

^b^ Compared with TP15.

^c^ Compared with TP25.

^d^ Compared with TP50.

* *p* < .001.

### 3.7 Evaluation of body weight

The differences in the body weights of mice after the administration of test samples were insignificant compared to the body weights before the treatments. In the toxicity group, the body weights of mice, after 28 days, were incomparable with their baseline body weights (Figure S1).

### 3.8 Blood lipid levels

**FIGURE 4:**
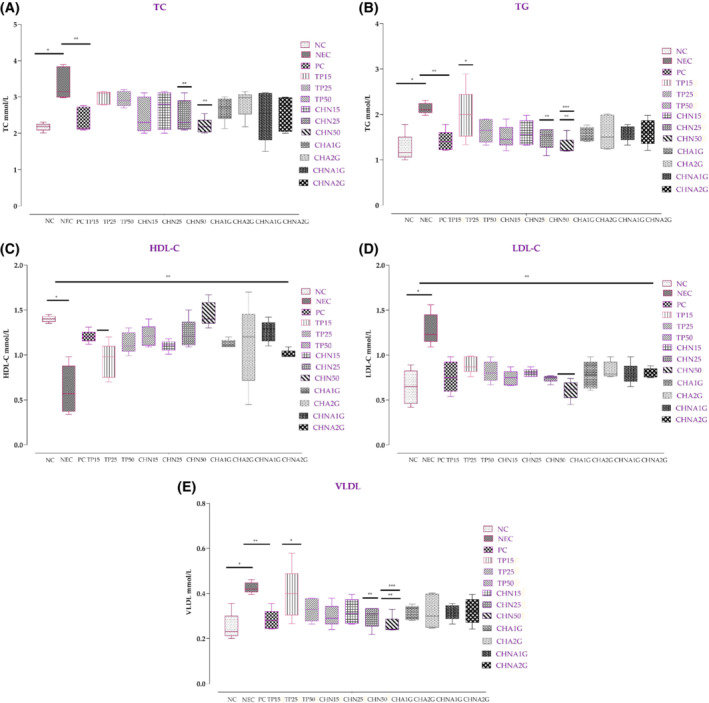
Evaluation of lipid levels in the blood: (A) total cholesterol, (B) total glycerides, (C) high‐density lipoproteins, (D) low‐density lipid proteins, and (E) very‐low‐density lipoproteins. *p‐value <.001 (NC compared with NEC). **p‐value <.001 (NEC compared with all treated groups). Values are presented in mean ± SD, one way ANOVA post hoc Tukey's test. CHA1G, extract 1000 mg/kg; CHA2G, extract 2000 mg/kg; CHN15, niosome group 15 mg/kg; CHN25, niosome group 25 mg/kg; CHN50, niosome group 50 mg/kg; CHNA1G, niosome 1000 mg/kg; CHNA2G, niosome 2000 mg/kg; NC, normal control; PC, positive contol; TP15, extract group 15 mg/kg; TP25, extract group 25 mg/kg; TP50, extract group 50 mg/kg.

Lipid levels examined in blood serum are presented in Figure 4. The results showed that TC levels were significantly high in alloxan‐induced diabetic mice as compared to normoglycemic mice (*p* < .001) (Figure 4A). The average value of TG in CHN25 and CHN50 showed comparable results (*p* < .05 & *p* < .01 respectively) with negative control (Figure 4B). While average HDL‐C values in normal control were 1.40 ± 0.03 mmol/L which drastically decreased in alloxan induced diabetic control group to 0.61 ± 0.26 mmol/L (Figure 4C). When compared with the negative control, the test groups; PC, TP25, TP50, CHN25 and CHN50 produced statistically significant results. The average LDL‐C values in NC were 0.64 ± 0.19 mmol/L which rose to 1.28 ± 0.07 mmol/L in NEC (*p* < .001) (Figure 4D). A significant decrease in average LDL‐C values were observed in PC 0.75 ± 0.17 mmol/L (*p* < .001) and CHN50 0.61 ± 0.10 mmol/L (*p* < .001) compared to diabetic control, after 28 days treatment. The average VLDL was 0.25 ± 0.06 mmol/L in NC, 0.42 ± 0.02 mmol/L in NEC but started to decline in PC (0.28 ± 0.04 mmol/L), TP50 (0.30 ± 0.05 mmol/L) and CHN50 (0.25 ± 0.03 mmol/L) (Figure 4E). In the acute toxicity group, lipid levels did not affect the health of the mice; the value was lower than NEC in CHA1G–CHNA2G.

### 3.9 Kidney function tests

**FIGURE 5:**
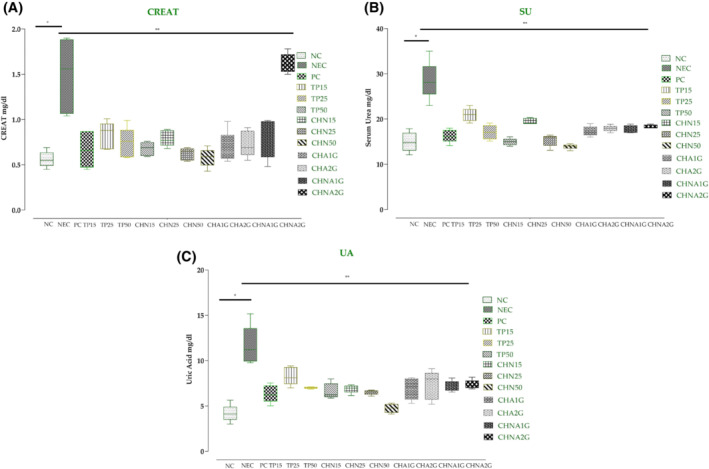
Evaluation of kidney biomarkers level in the blood: (A) serum creatinine levels, (B) serum urea levels, and (C) serum uric acid levels. *p‐value <.001 (NC compared with NEC). **p‐value <.001 (NEC compared with all treated groups). Values are presented in mean ± SD, one way ANOVA post hoc Tukey's test. CHA1G, extract 1000 mg/kg; CHA2G, extract 2000 mg/kg; CHN15, niosome group 15 mg/kg; CHN25, niosome group 25 mg/kg; CHN50, niosome group 50 mg/kg; CHNA1G, niosome 1000 mg/kg; CHNA2G, niosome 2000 mg/kg; NC, normal control; PC, positive contol; TP15, extract group 15 mg/kg; TP25, extract group 25 mg/kg; TP50, extract group 50 mg/kg.

The average serum creatinine levels in the NC group were 0.56 ± 0.08 mg/dL, values ranging from 0.45 to 0.58 mg/dL. While the average serum creatinine values of 14.95 ± 2.16 in the NEC group showed an increase with individual values in the range of 1.04 to 1.90 mg/dL. Intergroup analysis exhibited a significant decrease in all groups when compared with NEC, specifically, CHN50 0.58 ± 1.01 mg/dL (*p* < .001) and after 28 days of treatment came equivalent to NC. In the toxicity groups, CHNA2G resulted in the highest average CREAT value 1.47 ± 0.21 mg/dL, which gave comparable results with other groups; *p* < .001 (Figure 5A).

The average serum urea levels in normal control were 14.95 ± 2.16 mg/dL (range; 12.09–17.88 mg/dL). The average urea levels in negative diabetic mice rose to 28.49 ± 4.28 mg/dL (range; 22.98–35.01 mg/dL). Within the group, the negative control showed the highest value than the others (*p* < .001). The levels in TP50 were found out to be the same (14.95 ± 0.77 mg/dL) as in NC while CHN50 levels were 13.90 ± 0.56 mg/dL 28 days post‐treatment. The range of serum urea levels in toxicity groups was 17.30–18.31 mg/dL (Figure 5B).

The average serum uric acid levels in healthy normal control mice were 4.18 ± 0.94 mg/dL, which were higher in diabetic control mice; 11.64 ± 2.16 mg/dL. The average serum uric levels in the standard acarbose group were 6.35 ± 0.96 mg/dL—similar to TP50. While in the CHN50 group the average serum uric acid level was 4.74 ± 0.48 mg/dL, which was near to the NC group. In the toxicity group, the CHNA2G came out with higher levels 7.98 ± 0.511 mg/dL (Figure 5C).

### 3.10 Effect on hepatic biomarkers

**FIGURE 6:**
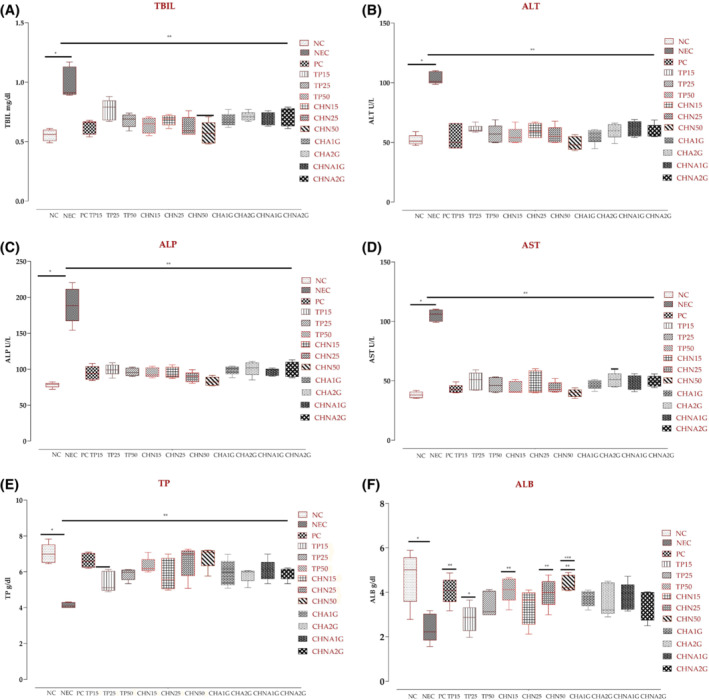
Evaluation of hepatic biomarkers level in the blood: (A) total bilirubin, (B) alanine transaminase, (C) alkaline phosphatase, (D) aspartate aminotransferase, (E) total protein, and (F) albumin. *p‐value <.001 (NC compared with NEC). **p‐value <.001 (NEC compared with all treated groups). ***p‐value <.05 (CHN50). Values are presented in mean ± SD, one way ANOVA post hoc Tukey's test. CHA1G, extract 1000 mg/kg; CHA2G, extract 2000 mg/kg; CHN15, niosome group 15 mg/kg; CHN25, niosome group 25 mg/kg; CHN50, niosome group 50 mg/kg; CHNA1G, niosome 1000 mg/kg; CHNA2G, niosome 2000 mg/kg; NC, normal control; PC, positive contol; TP15, extract group 15 mg/kg; TP25, extract group 25 mg/kg; TP50, extract group 50 mg/kg.

The average bilirubin levels in healthy mice were 0.554 ± 0.04 mg/dL. Elevated bilirubin levels were recorded in diabetic control mice (mean 0.99 ± 0.12 mg/dL), which decreased to 0.62 ± 0.06 mg/dL (*p* < .001) in acarbose treated mice. Among extract‐treated groups, TP50 showed average bilirubin levels of 0.638 ± 0.06 mg/dL which gave similar effect as of acarbose, while among niosome formulations; the CHN50 group had average bilirubin levels of 0.57 ± 0.09 mg/dL comparable to the normal control group. In the acute toxicity group, treated with CHNA2G, the average total bilirubin was 0.78 ± 0.08 mg/dL, while the minimum average of 0.68 ± 0.05 mg/dL was observed in CHA1G (Figure 6A).

Likewise, other hepatic biomarkers, such as ALT, ALP and AST exhibited higher levels in NEC (*p* < .001), precisely to be 103.88 ± 5.17, 189.11 ± 24.81 and 105.05 ± 5.00 U/L respectively. In the NC group, the mean values of ALT, ALP, and AST were 51.96 ± 4.38, 78.12, and 38.14 ± 2.71 U/L, respectively. After treatment with the extracts (15, 25 and 50 mg/kg) the average values of ALT were recorded as 61.15 ± 3.39, 57.04 ± 7.83 and 55.24 ± 7.02 U/L respectively. Likewise, average values of ALP in extracts (15, 25, and 50 mg/kg) were 99.5 ± 7.84, 96.14 ± 5.60 and 95.10 ± 6.80 U/L, respectively, and of AST were 49.76 ± 7.53, 46.69 ± 6.31 and 44.03 ± 5.26 U/L, respectively. The intergroup analysis of each test revealed that the results of ALT, ALP, and AST were statistically significant (*p* < .001) when compared with their alloxanized groups. Similarly, treatment with niosomal formulations resulted in a significant reduction in hepatic biomarkers compared to NEC (*p* < .001), particularly CHN50 that exhibited average ALT, ALP, and AST values; 49.22 ± 5.71, 83.12 ± 6.18, and 39.70 ± 3.34 U/L, respectively. Intergroup examination revealed that toxicity tested groups CHA1G to CHNA2G also indicated that values of ALT, ALP, and AST were significantly lower (*p* < .001) than the NEC group (Figure 6B–D).

The average total protein levels in the normal control group were 7.01 ± 0.55 g/dL (range 6.45 to 7.83) with mean albumin levels of 4.66 ± 1.17 g/dL (range 3.78 to 5.89) which seemingly decreased in alloxan‐induced diabetic mice; average TP levels 4.13 ± 0.16 g/dL and ALB levels 2.39 ± 0.64 g/dL. Oral administration of acarbose resulted in increased average TP and albumin levels to 6.63 ± 0.40 and 4.07 ± 0.61 g/dL when compared with the NEC group. Out of 3 extract treated groups (TP15, TP25, and TP50), only TP50 showed a significantly high effect on average TP and albumin levels, that is, 6.33 ± 0.43 g/dL and ALB 4.12 ± 0.57 g/dL, when compared with the negative group. Furthermore, niosomes treatment resulted in a remarkable increase in TP and ALB in diabetic mice (*p* < .001), explicitly by CHN50; TP 6.83 ± 0.60 g/dL and ALB 4.39 ± 0.36 g/dL. In CHA1G group the TP and ALB were 5.94 ± 0.73 g/dL (range 5.09–6.98 g/dL) and 3.74 ± 0.36 g/dL (range 3.20–4.10 g/dL), respectively, while in CHA2G the TP and ALB were 5.81 ± 0.39 g/dL (range 5.12–6.07 g/dL) and 3.63 ± 0.74 g/dL (range 2.90–4.50 g/dL) respectively. Moreover, in CHNA1G group, the mean TP and ALB levels were 6.07 ± 0.59 g/dL (range 5.34–6.99 g/dL) and 3.82 ± 0.62 g/dL (range 3.16–4.72 g/dL), respectively, while in CHNA2G group, the mean TP and ALB levels were 5.92 ± 0.35 g/dL (range 5.34–6.21 g/dL) and 3.49 ± 0.71 g/dL (range 2.49–4.02 g/dL) respectively (Figure 6E,F).

### 3.11 Organ to body weight ratio

Consecutive treatment of extract and phytoniosome along with standard acarbose in alloxanized mice did not show significant changes in relative organ body weights. The average weight of the liver relative to the % body weight of the NEC group was significantly increased when compared with NC, treated groups and acute toxicity groups (*p* < .05 & *p* < .001).

The average size of the pancreas of the diabetic control group was larger than the other groups; including acute toxicity groups. However, intergroup analysis of treated groups (extracts and niosomes) showed no prominent change in the relative size of the pancreas. No difference in the relative weights of the kidney was found (Table S1).

### 3.12 Histopathology of organs

**FIGURE 7:**
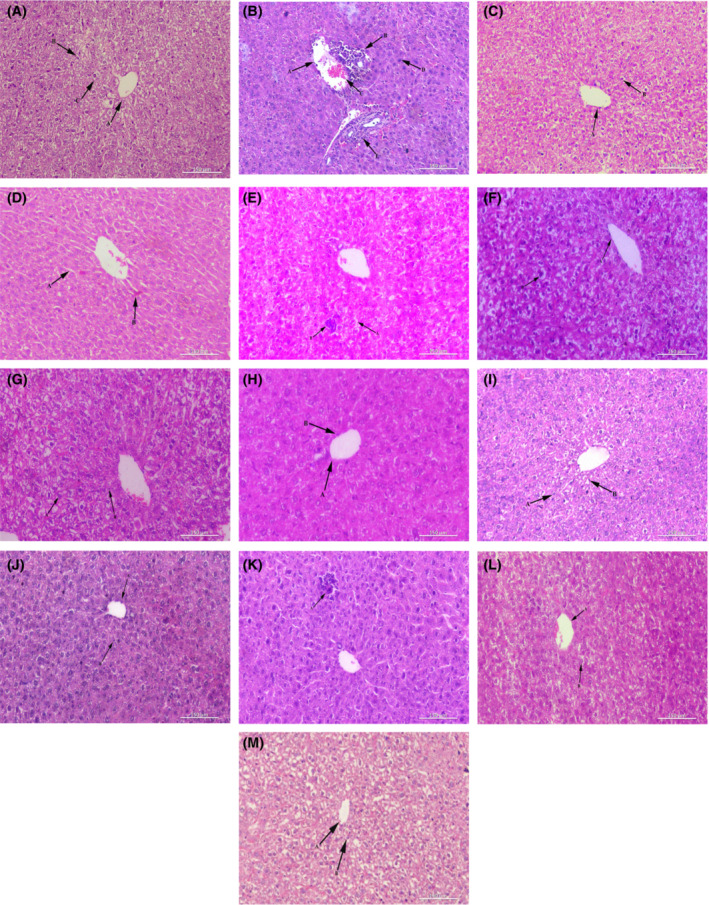
Histopathology of mice liver 28 days post treatment using light micrographs. (A) Normal healthy liver: A—proper central vein, B—radially arranged hepatocytes, C—sinusoidal spaces. (B) Diabetic alloxanized liver: A—irregular shape of central vein, B—shrunken, dark apoptotic hepatic cells, C—focal necrosis, D—abnormal arrangement of hepatocytes in sinusoid, E—infiltration of inflammatory cell. (C) treated with acarbose: A—Central vein with sharp boundaries, B—arranged liver cells. (D) TP15 treated, A—wide sinusoidal spaces, B—necrotic foci. (E) TP25 treated: A—cytoplasmic degeneration, B—accumulation of cells. (F) TP50 treated: A—central vein with sharp boundary, B—binucleated cells. (G) CHN15 treated: A—arranged binucleated cells, B—Von Kupffer cells. (H) CHN25 treated: A—proper shape of central vein, B—arranged hepatic cells. (I) CHN50 treated: A—binucleated cells are present, B—Kupffer cells are arranged, (J) Acute toxicity bearing CHA1G: A—binucleated cells present, B—Kupffer cells are present. (K) Acute toxicity bearing CHA2G: A—inflammatory cells visible. (L) Acute toxicity bearing CHNA1G: A—central vein with sharp boundaries, B—liver cells were arranged radially. (M) Acute toxicity bearing CHNA2G: A—proper central vein, B—binucleated cells were observed. CHA1G, extract 1000 mg/kg; CHA2G, extract 2000 mg/kg; CHN15, niosome group 15 mg/kg; CHN25, niosome group 25 mg/kg; CHN50, niosome group 50 mg/kg; CHNA1G, niosome 1000 mg/kg; CHNA2G, niosome 2000 mg/kg; NC, normal control; PC, positive contol; TP15, extract group 15 mg/kg; TP25, extract group 25 mg/kg; TP50, extract group 50 mg/kg.

**FIGURE 8:**
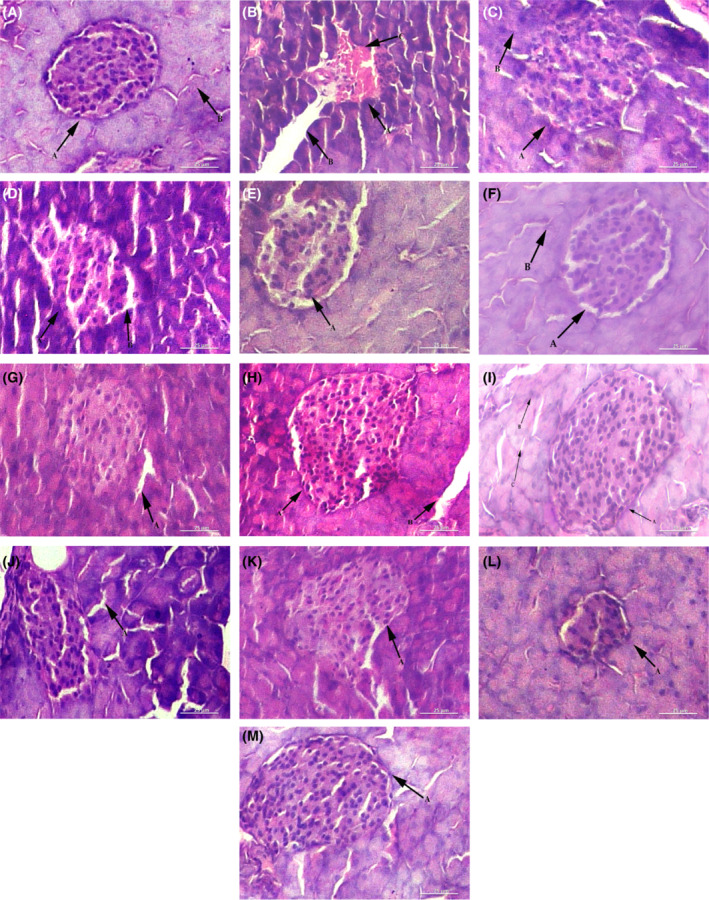
Photomicrographs of histopathology of mice pancreas 28 days post treatment using light micrographs. (A) Normal healthy pancreas: A—normal islets of Langerhans, B—healthy exocrine portion. (B) Diabetic mouse: A—degenerated islets of Langerhans, B—vacuolated cytoplasm, C—focal necrosis. (C) Treated with acarbose: A—normal islets of Langerhans, B—exocrine portion looked normal. (D) TP15 treated: A—inflammation, B—large cytoplasmic spaces. (E) TP25 treated: A—normal islets of Langerhans. (F) TP50 treated: A—normal islets of Langerhans, B—normal exocrine portion. (G) CHN15 treated: A—irregular cytoplasmic space. (H) CHN25 treated: A—healthy islets of Langerhans, B—wide cytoplasmic space. (I) CHN50 treated: A—normal islets of Langerhans, B—healthy exocrine portion, C—normal cytoplasmic region. (J) Acute toxicity bearing CHA1G: A—wide sinuses (K) Acute toxicity bearing CHA2G, irregularislets of Langerhans. (L) Acute toxicity bearing CHNA1G: A—normal islets of Langerhans. (M) Acute toxicity bearing CHNA2G: A—healthy islets of Langerhans. CHA1G, extract 1000 mg/kg; CHA2G, extract 2000 mg/kg; CHN15, niosome group 15 mg/kg; CHN25, niosome group 25 mg/kg; CHN50, niosome group 50 mg/kg; CHNA1G, niosome 1000 mg/kg; CHNA2G, niosome 2000 mg/kg; NC, normal control; PC, positive contol; TP15, extract group 15 mg/kg; TP25, extract group 25 mg/kg; TP50, extract group 50 mg/kg.

**FIGURE 9:**
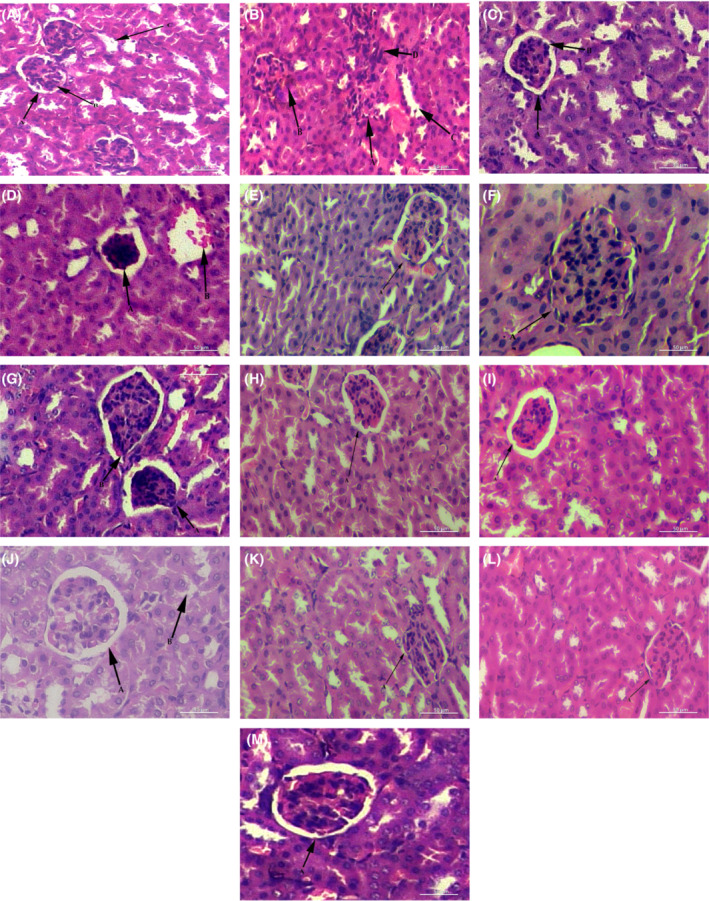
Photomicrographs of histopathology of mice kidney 28 days post treatment using light micrographs. (A) Normal healthy kidney: A—normal glomerulus, B—normal Bowman's capsule, C—clear renal tubules. (B) Diabetic mouse kidney: A—degenerative glomerulus, B—necrosis, C—wide cytoplasmic region, D—diffused muscular media. (C) Treated with acarbose: A—normal glomerulus, B—normal Bowman's capsule. (D) TP15 treated: A—damaged Bowman's capsule, B—glomerulosclerosis. (E) TP25 treated: A—degenerated Bowman's capsule. (F) TP50 treated: A—irregular Bowman's capsule. (G) CHN15 treated: A—infiltration from glomerulus, B—deformed Bowman's capsule. (H) CHN25 treated: A—normal glomerulus and Bowman's capsule: (I) CHN50 treated: A—rejuvenated Bowman's capsule. (J) Acute toxicity bearing CHA1G: A—normal glomerulus, B—healthy renal tubules. (K) Acute toxicity bearing CHA2G: A—congested glomerulus. (L) Acute toxicity bearing CHNA1G: A—degenerated Bowman's capsule. (M) Acute toxicity bearing CHNA2G: A—normal glomerulus. CHA1G, extract 1000 mg/kg; CHA2G, extract 2000 mg/kg; CHN15, niosome group 15 mg/kg; CHN25, niosome group 25 mg/kg; CHN50, niosome group 50 mg/kg; CHNA1G, niosome 1000 mg/kg; CHNA2G, niosome 2000 mg/kg; NC, normal control; PC, positive contol; TP15, extract group 15 mg/kg; TP25, extract group 25 mg/kg; TP50, extract group 50 mg/kg.

Examination of mice organs revealed visible pathological changes in the NEC. After treatment, TP50 and CHN50 exhibited normal cells as of NC (Figures 7, 8, 9).

### 3.13 GC–MS analysis

The GC–MS analysis of chloroform extract of leaves of *T. pallida* recorded 21 metabolites. The results revealed the presence of seven hydrocarbons, seven fatty acids, and their esters (four saturated and three unsaturated), two terpenes, two sterols, one phenolic compound, one flavonoid, and one delta‐lactam. The chemical name, retention time, and % area of putative compounds is presented in Table 5. The highest percentage of peak area was covered by oleic acid (10.01%) followed by stigmasterol (9.61%) and hentriacontane (5.04%).

**TABLE 5:** Secondary metabolites identified by GC–MS of chloroform extract of leaves of *Tradescantia pallida*.

Putative compounds	Molecular formula	Molar mass (g/mol)	Retention time (min)	Area (%)
Tetradecane	C_14_H_30_	198.39	17.29	0.03
2,4‐Di‐tert‐butylphenol	C_14_H_22_O	206.32	19.29	0.83
1‐Hexadecene	C_16_H_32_	224.42	20.19	0.04
Hexadecane	C_16_H_34_	226.41	20.31	0.12
Spathulenol	C_15_H_24_O	220.35	22.12	0.22
2‐Piperidinone, N‐[4‐bromo‐n‐butyl]‐	C_9_H_16_BrNO	234.13	24.33	0.52
Caryophyllene	C_15_H_24_	204.35	25.13	0.06
Oleic acid	C_18_H_34_O_2_	282.50	26.24	10.01
Nonadecanoic acid	C_19_H_38_O_2_	298.48	28.19	0.05
Linoleic acid	C_18_H_32_O_2_	280.40	29.24	0.11
Palmitic acid	C_16_H_32_O_2_	256.42	31.34	2.15
Methyl linolenate	C_19_H_32_O_2_	292.50	31.49	1.02
7,9‐Di‐tert‐butyl‐1‐1‐oxaspiro(4,5)deca‐6,9‐diene‐2,8‐dione	C_17_H_24_O_3_	276.40	31.66	1.72
Methyl palmitate	C_17_H_34_O_2_	270.50	31.85	2.29
Hexacosane	C_26_H_54_	366.71	32.89	0.13
Heptacosane	C_27_H_56_	380.74	33.91	1.53
Octacosane	C_28_H_58_	394.77	34.31	3.10
Methyl tricosanoate	C_24_H_48_O_2_	368.70	36.08	0.66
Hentriacontane	C_31_H_64_	436.80	38.77	5.04
Stigmasterol	C_29_H_48_O	412.69	42.69	9.61
Sitosterol	C_29_H_50_O	414.68	48.64	2.56

### 3.14 ADME profile of GC–MS identified active compounds

Absorption, distribution, metabolism, and excretion profile of the GC–MS identified compounds of *T. pallida* leaves were predicted to assess the pharmacokinetics, pharmacodynamics and drug‐likeness parameters. All the compounds exhibited compliance with the standard range of Lipinski's rule of five and Jorgensen's rule of three (Table 6).

**TABLE 6:** ADME profile of GC–MS identified active compounds.

Molecules	Molecular weight	QPlogHERG	QPPCaco	QPlogBB	QPPMDCK	QPlogKp	QPlogKhsa	Percent human oral absorption	Rule of five	Rule of three
2,4‐Di‐tert‐butylphenol	206.327	−3.278	4802.148	0.116	2696.126	−1.616	0.545	100	0	0
Spathulenol	220.354	−2.992	5093.648	0.263	2874.515	−1.876	0.601	100	0	0
2‐Piperidinone, N‐[4‐bromo‐n‐butyl]‐	234.135	−1.912	2293.457	0.182	6223.504	−1.868	−0.626	100	0	0
Caryophyllene	204.355	−3.000	9906.038	1.032	5889.293	−1.394	0.954	100	1	1
Oleic acid	282.465	−3.522	240.863	−1.541	135.069	−1.977	0.668	89.922	1	1
Nonadecanoic acid	298.508	−3.573	235.395	−1.705	131.757	−1.885	0.781	92.196	1	1
Linoleic acid	280.450	−3.408	294.559	1.343	167.891	−1.837	0.606	90.580	1	0
Palmitic acid	256.428	−3.059	235.395	−1.426	131.757	−2.173	0.410	100	0	0
Methyl linolenate	292.461	−5.150	2954.883	−0.807	1595.689	−1.095	0.998	100	1	1
7,9‐Di‐tert‐butyl‐1‐1‐oxaspiro(4,5)deca‐6,9‐diene‐2,8‐dione	276.375	−3.258	2050.105	−0.159	1074.838	−2.649	−0.037	100	0	0
Methyl palmitate	270.454	−5.154	2954.882	−0.893	1595.689	−1.196	0.837	100	1	1
Methyl tricosanoate	368.642	−6.027	3021.100	−1.397	1634.375	−0.506	1.693	100	1	1
Stigmasterol	412.698	−4.246	3466.921	−0.251	1896.555	−1.724	1.903	100	1	1
Sitosterol	414.713	−4.590	3419.737	−0.342	1868.671	−1.644	2.015	100	1	1

### 3.15 Molecular docking

**FIGURE 10:**
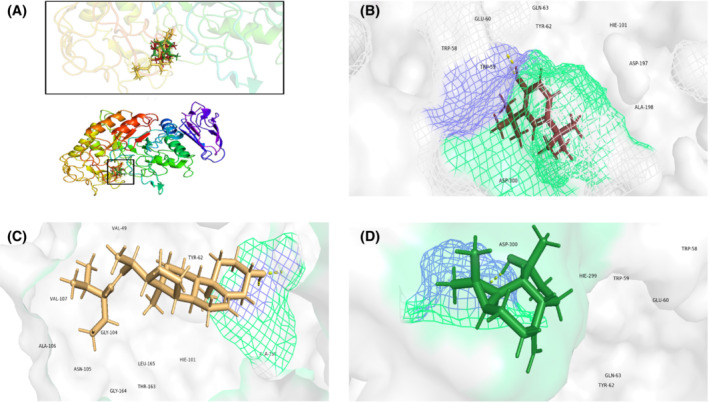
Molecular docking of active compounds of *Tradescantia pallida* leaves. (A) Bull‐shaped human α‐amylase protein 4GQR with docked compounds in the same binding pocket. (B) 2,4‐Di‐tert‐butylphenol docked with 4GQR. (C) Stigmasterol docked with 4GQR. (D) Spathulenol docked with 4GQR. Yellow dotted lines represent hydrogen bonding.

**FIGURE 11:**
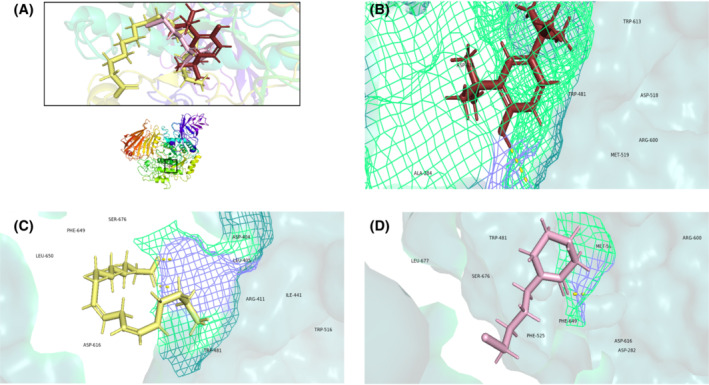
Molecular docking of active compounds of *Tradescantia pallida* leaves. (A) Hen‐shaped human α‐glucosidase protein 5NN5 with docked compounds in the same binding pocket. (B) 2,4‐Di‐tert‐butylphenol docked with 5NN5. (C) Linoleic acid docked with 5NN5. (D) 2‐Piperidinone, N‐[4‐bromo‐n‐butyl]‐ docked with 5NN5. Yellow dotted lines represent hydrogen bonding.

In silico molecular docking was performed on GC–MS identified compounds. The first three compounds that showed the highest docking score with protein 4GQR and 5NN5 are presented in Table 7. The bull‐shaped protein 4GQR with the docked compounds is presented in Figure 10A. The best scoring pose of the compounds 2,4‐Di‐tert‐butylphenol, stigmasterol and spathulenol with α‐amylase protein 4GQR are represented in Figure 10B–D. Likewise the hen‐shaped protein 5NN5 depicting docked compounds are shown in Figure 11A and the best scoring pose of 2,4‐Di‐tert‐butylphenol, linoleic acid and 2‐Piperidinone, N‐[4‐bromo‐n‐butyl]‐ with α‐glucosidase are shown in Figure 11B–D. From the docking analysis, we found that the highly active compounds of 4GQR have shown maximum binding affinity at −5.958, −4.731 and −4.232 Kcal/mol while of 5NN5 at −3.880, −3.593 and −3.442 Kcal/mol. Energy minimization studies were carried out using Prime molecular mechanics‐generalized Born surface area (Prime MM‐GBSA). The ΔG bind value of the active compounds of 4GQR was in the range of −38.63 to −20.29 and that of 5NN5 were from −25.62 to −7.18 (Table 7).

**TABLE 7:** Molecular docking and Prime MM‐GBSA calculations of GC–MS identified active compounds.

Ligand	Docking score	Glide energy	Glide score	Hydrogen bonding	Hydrogen bond distance Å	MM‐GBSA ΔG bind
*4GQR*						
2, 4‐Di‐tert‐butylphenol	−5.958	−21.899	−5.958	GLN63‐ H, ligand O; TRP59‐ O, ligand H	2.1, 1.9	−20.29
Stigmasterol	−4.731	−32.329	−4.731	ASP197‐ O, ligand H; ARG195‐ H, ligand O	1.8, 2.1	−38.63
Spathulenol	−4.232	−21.739	−4.232	THR163‐ O, ligand H	2.3	−22.68
*5NN5*						
2, 4‐Di‐tert‐butylphenol	−3.880	−22.383	−3.880	ASP282‐O, ligand‐ H	2.3	−25.62
Linoleic acid	−3.593	−26.310	−3.596	ARG411‐ H, ligand‐ O	1.7, 1.7	−16.86
2‐Piperidinone, N‐[4‐bromo‐n‐butyl]‐	−3.442	−20.145	−3.442	ARG600‐H, ligand‐ O	2.0	−7.18

## 4 DISCUSSION

Despite the availability of the already marketed antidiabetic drugs, herbal medicines are still considered less toxic and more efficacious than synthetic ones.^52^, ^53^ Natural drugs have been successfully utilized by the scientific community for the discovery of new drugs and have exhibited far‐reaching impact in the field of chemicobiology.^54^, ^55^, ^56^ The qualitative and quantitative analysis of chloroform extract of leaves of *T. pallida* revealed the presence of polyphenols, flavonoids, lipids and proteins, which in accordance with the previous studies are excellent therapeutic agents against various deadly diseases.^57^, ^58^, ^59^, ^60^, ^61^ The UV–visible profile showed the characteristic peaks of phenols, flavonoids, and alkaloids^62^, ^63^ in the UV region and predicted the presence of terpenoids, chlorophyll, and tannins in the visible region.^63^, ^64^ FTIR analysis depicted the presence of phenols, alkanes, carbohydrates,^65^, ^66^ lipid acyl chains,^65^, ^67^ isoprenoids and amines.^68^ Despite being the active source of therapy, there are a few limitations associated with the use of herbal medicines. The major ones are the delivery of the large size of secondary metabolites in the herbal drugs, dose regimen including size and frequency of dosage, and achievement of highest concentration at the site of action.^69^ To overcome the issues, we aimed to synthesize niosomes using plant crude extract. We comprehensively compared and evaluated the antidiabetic activity of *T. pallida* leaves crude extract with those of phytoniosomes prepared with chloroform extract of *T. pallida* leaves.

The entrapment efficiency of phytoniosomes was up to 89.12%, with small particle sizes and low PDI value. The ability of smaller particle sizes to more effectively entrap the medicine is a well‐known phenomenon. This might be the result of the high concentration of Span 60 in the mixture, which was consistent with past research demonstrating Span 60's key involvement in the effective drug loading.^70^

Drug delivery with the desired properties depends on a number of factors, including drug release rate. It depends on a number of variables, including as the drug's characteristics, the size and shape of the niosomes, the system's monodispersity (PDI), and the inclusion of additional ingredients like surface active agents.^71^ The formulation of phytoniosomes used in this investigation has a moderate quantity of cholesterol. The presence of cholesterol is critical for drug entrapment because it helps turn gel state bilayers into liquid state bilayers, increasing drug entrapment and regulating drug release.^72^ The niosomal formulations' in vitro drug release was biphasic, with an initial brief burst of release followed by a prolonged controlled release phase lasting up to 12 h. Pure extract, in comparison, released all of the medication in 4 h. Typically, the desorption of extract from the niosome surface layer occurs during the burst release phase. The burst release, however, was limited, indicating that the majority of the medication was contained within the niosomes. After the drug was surface desorbed and released, the remaining extract diffused across the niosomes' surface layers during the slow release phase.^73^

The antidiabetic potential of chloroform extract and phytoniosomes of leaves of *T. pallida* was evaluated by implying various animal models. In the acute toxicity study, no mortality was recorded at 2000 mg/kg dose of both extract and phytoniosmes. According to OECD guidelines and Loomis's Toxicology testing substances with LD50 values, 2000 mg/kg or higher are considered safe to use.^74^, ^75^

Acarbose is a carbohydrate, specifically an oligosaccharide, which acts by reversibly inhibiting the α‐amylase and α‐glucosidase enzyme in the intestine. These enzymes play a pivotal role in the digestion of carbohydrates into their smaller units, monosaccharides.^76^ However, there are many side effects associated with the prolonged use of acarbose, such as flatulence, abdominal distention, and increased rate of bacterial infections.^77^ Hence, the medicinal plants that could safely inhibit the α‐amylase and α‐glucosidase are of much importance in the search for novel antidiabetic agents.^78^, ^79^ The observed hypoglycemic effect after treatment with extract and phytoniosome may be due to inhibition of α‐amylase and α‐glucosidase thus attenuation of postprandial glucose absorption in the blood.^80^

The oral glucose loaded test is used to analyze the body's potential to utilize glucose. This is an acceptable model to detect patients that are on the borderline to have diabetes.^81^, ^82^ Statistically, no significant results were seen in the PC group in the oral glucose‐loaded test when compared with the baseline, likewise, TP15, TP25, and CHN25 could not exert comparable results concerning their baselines. It took 90 min to lower the blood glucose level in TP50 that lasted a long time, till 120 min. Contrary to this CHN25 and CHN50 showed peak hypoglycemic effect at 60 min (*p* < .001) that prolonged till the end of the study. The observed hypoglycemic effect may be due to inhibition of α‐amylase thus attenuation of postprandial glucose absorption in the blood. The improved glucose tolerance may also be linked with glycogenesis in the liver, decreased gluconeogenesis, and enhanced glucose utilization by tissues.^83^, ^84^

Induction of diabetes in animal models is usually persuaded by Alloxan. The collected evidence suggests that alloxan works by rapidly depleting β cells through DNA alkylation and cytotoxic free radicals that result in the inflammation of the islet of Langerhans that progresses to accumulation of macrophages and lymphocytes. These changes in pancreas lead to decreased level of insulin in plasma and hence stable hyperglycemic state.^85^, ^86^ In this study, alloxan 150 mg/kg was used to induce the hyperglycemic state, after 72 h blood glucose level of mice above 250 mg/dL was considered diabetic.

The results indicate that prolonged use of CHN50 can prevent the death of β‐cells and lead to the proliferation of normal healthy cells. The effect of TP50 alone can be compared with CHN25 and CHN50 that declare the positive impact of niosomes encapsulating crude extract.

The bodyweight in normal healthy mice increased by the end of study, however, diabetic control negative groups exhibited decreased body weight. The substantial drop in weight was concomitant with the high blood glucose level in mice. This loss is attributed to the high catabolism of structural proteins and fats, which served as a source of energy in the absence of carbohydrates.^87^ However, treatment with acarbose, TP50, CHN25, and CHN50 improved the body weight along with the stabilization of high blood glucose levels. This protective effect of extract and niosome on body weight could be due to their blood glucose‐lowering effect.^88^

The biochemical analysis of lipids revealed that the levels of TC, TG, HDL‐C, LDL‐C, and VLDL were incredibly high in diabetic control mice as compared to normal healthy mice. While the treated groups showed less damage especially CHN50 whose profile was similar to the NC group. Insulin causes the metabolism of lipids through lipoprotein‐lipase enzyme activation which aids in the breakdown of triglycerides into glycerol and fatty acids. These fatty acids play a pivotal role by providing energy to the body and acting as a storage carrier. In diabetes insulin deficiency or resistance inactivates the lipoprotein‐lipase enzyme that leads to hypertriglyceridemia.^89^, ^90^ Elevated LDL‐C is due to the transportation of cholesterol to the other tissues from the liver that could develop coronary heart disease.^91^ While HDL‐C is appraised as good cholesterol and valuable lipoprotein prevents toppling of cholesterol by transporting cholesterol esters and endogenous cholesterol to the steroidogenic cells and liver, thus preventing atherosclerosis.^92^ The study revealed that niosome treated alloxan‐induced diabetic mice had normal lipid values when compared with extract‐treated and acarbose treated mice.

The kidney maintains the chemical composition of fluids in the body by acidifying the urine and removing toxic metabolic wastes including creatinine, urea, and uric acid. In renal diseases accompanying metabolic disorders, the levels of these metabolites increase in the blood.^93^ In diabetic control mice, the concentrations of serum creatinine, serum urea, and serum uric acid were significantly higher as compared to normal healthy and CHN50 treated animals. The high rate of release of purine and elevated activity of xanthine oxidase may be the cause of the increased concentration of these metabolites in the blood. The metabolic disturbance in the diabetic state leads to increased activities of lipid peroxidation, xanthine oxidase, urea, and creatinine that cause ketosis that progresses to acidosis.^94^, ^95^ The maintained levels of kidney biomarkers were observed in experimental mice treated with extract‐loaded niosomal formulation CHN50.

The liver is the main organ that provides first‐pass metabolism to the orally administered drugs and changes in the liver tissues caused by these orally administered pharmacological agents may result in elevated levels of TBIL, ALT, ALP, and AST. Possibly, the increased levels of bilirubin in most of the treated groups were due to the destruction or mistreatment of hemoglobin by the liver.^96^ Likewise, total protein levels are one of the indicators to judge liver function as most of the proteins are synthesized in the liver and damaged liver will reduce the synthesis of protein.^91^ The level of protein and albumin was restored in CHN50, this could be due to the active transport of amino acids through the cell that stimulated the protein production.^97^

Researchers frequently apply organ to body weight ratio as an indicator to assess normal organs. The organ size disturbs when chemicals or toxins are ingested.^98^ After treatment, the relative organ body weight was measured and concluded that percentage relative weight was changed in NEC, TP15, and CHN15, however, the rest of the groups had almost similar organ weights when compared to NC that reflected the non‐hazardous and non‐deleterious impact of treatment in mice. The prominent increment or decrement in the organ to body ratio indicates the effects induced by drugs on the visceral organs.^99^ The change in liver weight may be due to decreased detoxification of toxic substances; likewise, kidney weight serves as a tool to analyze the anatomy and excretion property which is an indirect effect of an ineffective liver.^99^, ^100^ Niosomal treatment abbreviated the organ to body weight ratio; indicating a reversal of organ hypertrophy in alloxan‐induced diabetic mice.

It was observed from the histological images that oral treatment of extract‐loaded niosomes protects the body organs efficiently. The histopathological data achieved from this study strongly recommends that crude extract encapsulated in niosome can be a valuable adjuvant therapy to lower the blood glucose level in diabetes, as well as it may reduce the onset of secondary complications associated with diabetes, however, further investigations are required.

GC–MS analysis of chloroform extract of *T. pallida* leaves identified potent antidiabetic metabolites that have never been reported earlier from this plant. One of the main metabolites is oleic acid, which has been reported to have a protective effect on β‐cells with increased insulin secretion. It also enhances mitochondrial biogenesis and prevents insulin resistance and diabetes type 2.^101^, ^102^, ^103^ Another important metabolite is stigmasterol that has exhibited exceptional antidiabetic activity by inhibiting α‐amylase, postprandial glucose level, reduced creatinine and urea level, and increased insulin uptake from pancreatic cells.^104^, ^105^, ^106^, ^107^, ^108^ Previous studies have reported that sitosterol is an excellent source to reduce blood glucose level and hyperlipidemia associated with diabetes.^109^, ^110^, ^111^, ^112^, ^113^ Linoleic acid has shown to improve glucose uptake by activating AMP‐activated protein kinase pathway and regulated insulin action by inhibiting protein tyrosine phosphorylation.^114^ While caryophyllene has been reported to increase insulin secretion, glucose uptake and reduced glucose absorption. Palmitic acid has also shown improved blood glucose levels in db/db mice model.^115^ In addition, caryophyllene has proven to decrease the levels of cholesterol and triglycerides, increased oxidation of fatty acids and maintained lipid levels with hypolipidemic effects.^116^, ^117^, ^118^, ^119^, ^120^, ^121^, ^122^ Spathulenol has proven to have antinociceptive, anti‐inflammatory, and anticancer potential.^123^, ^124^, ^125^ 2, 4‐Di‐tert‐butylphenol has shown antioxidant, anti‐inflammatory, cytotoxic, antibacterial, antiviral, and antifungal activities.^126^, ^127^, ^128^, ^129^, ^130^, ^131^, ^132^ No antidiabetic activity has been reported for spathulenol, 2, 4‐Di‐tert‐butylphenol and 2‐Piperidinone, N‐[4‐bromo‐n‐butyl]‐. Thus, our study is novel in identifying the in silico antidiabetic potential of spathulenol, 2, 4‐Di‐tert‐butylphenol and 2‐Piperidinone, N‐[4‐bromo‐n‐butyl]‐ against α‐amylase and α‐glucosidase inhibition.

ADME profile plays a key role in understanding the drug‐like behavior of compounds in medicinal chemistry.^133^ Our study showed that all GC–MS identified compounds of *T. pallida* have a high potential to be good drug candidates.

Molecular docking is a reliable computer‐based methodology to evaluate the potent metabolites and accelerates the drug design process by revealing the interactions of receptor and ligand, energies and binding mode.^134^ Likewise, the Prime MM‐GBSA technique is used to calculate the binding free energies to corroborate the docked complexes.^135^ Our study suggested that 2, 4‐Di‐tert‐butylphenol, stigmasterol, spathulenol, linoleic acid and, 2‐Piperidinone, N‐[4‐bromo‐n‐butyl]‐ have high binding affinities with human α‐amylase and α‐glucosidase proteins. The interaction could be attributed to the hydrogen bonds formation. Similarly, according to our Prime MM‐GBSA data, stigmasterol, 2, 4‐Di‐tert‐butylphenol and spathulenol showed the most negative energies which unveils the stability of the protein‐ligand complexes. Our study is novel in evaluating the molecular docking and Prime MM/GBSA calculations on 2, 4‐Di‐tert‐butylphenol, stigmasterol, spathulenol, linoleic acid and, 2‐Piperidinone, N‐[4‐bromo‐n‐butyl]‐ against the 4GQR and 5NN5 targets and anticipates their antidiabetic potential.

The antihyperglycemic effect and antidiabetic potential exhibited by crude extract of *T. pallida* can be attributed to its potent secondary metabolites.^136^, ^137^, ^138^, ^139^ The results concur findings of earlier studies performed on Tradescantia species.^12^, ^13^ Previous studies have revealed that niosomes have shown better antidiabetic potential as compared to their parent extract, due to better stability, enhanced absorption, and sustained release.^41^, ^140^, ^141^, ^142^, ^143^ The extraordinary positive effect of phytoniosome of *T. pallida* in ameliorating diabetes and its complications are due to the amphiphilic nature of the niosomal complex with crude extract, which eventually enhanced the lipid and water miscibility of phytochemical constituents of *T. pallida* leaves.

## 5 CONCLUSIONS

In conclusion, the present study collected adequate data that indicates niosomes loaded with extract have better antidiabetic effect than the crude extract and standard drug. This study reveals the ameliorative impact of crude extract and extract‐loaded phytoniosomes on diabetic mice by maintaining the blood glucose levels and has shown to ameliorate diabetes‐related complications. It can be concluded that phytoniosome prepared from chloroform extract of leaves of *T. pallida* at a dose of 50 mg/kg is remarkably effective against alloxan‐induced diabetes. Molecular docking studies attributed the antidiabetic effect to 2, 4‐Di‐tert‐butylphenol, stigmasterol, spathulenol, linoleic acid, and 2‐Piperidinone, N‐[bromo‐n‐buty] present in the extract of leaves by inhibiting α‐amylase and α‐glucosidase.

## AUTHOR CONTRIBUTIONS

Conceptualization: Fariha Imtiaz, Muhammad Islam, Hamid Saeed, and Hassaan Anwer Rathore. Methodology: Fariha Imtiaz, Muhammad Islam, Hamid Saeed, and Hassaan Anwer Rathore. Software: Fariha Imtiaz, Abrar Ahmed, and Hamid Saeed. Validation: Fariha Imtiaz, Abrar Ahmed, and Hassaan Anwer Rathore. Formal analysis: Fariha Imtiaz, Muhammad Islam, and Hassaan Anwer Rathore. Investigation: Fariha Imtiaz, Muhammad Islam, and Hamid Saeed. Data curation: Fariha Imtiaz, Muhammad Islam, and Hamid Saeed. Writing—original draft preparation: Fariha Imtiaz, Muhammad Islam, and Hamid Saeed. Writing—review and editing: Fariha Imtiaz, Muhammad Islam, and Hassaan Anwer Rathore. Supervision: Muhammad Islam, Hamid Saeed, and Abrar Ahmed. Funding acquisition: Hassaan Anwer Rathore and Muhammad Islam. All the authors have read and agreed to the published version of the manuscript.

## DISCLOSURES

The authors declare no conflict of interest.

## Supporting information


Figure S1

Appendix S1

## Data Availability

This manuscript is based on original data generated in our labs and can be made available from authors on reasonable request.
