# Supplementary figures and images for: Assessment of the antidiabetic potential of extract and novel phytoniosomes formulation of Tradescantia pallida leaves in the alloxan‐induced diabetic mouse model
Source: FASEB J. 2023 Mar 1;37(4):e22818. doi: 10.1096/fj.202201395RR (PMC11977607; doi:10.1096/fj.202201395RR)

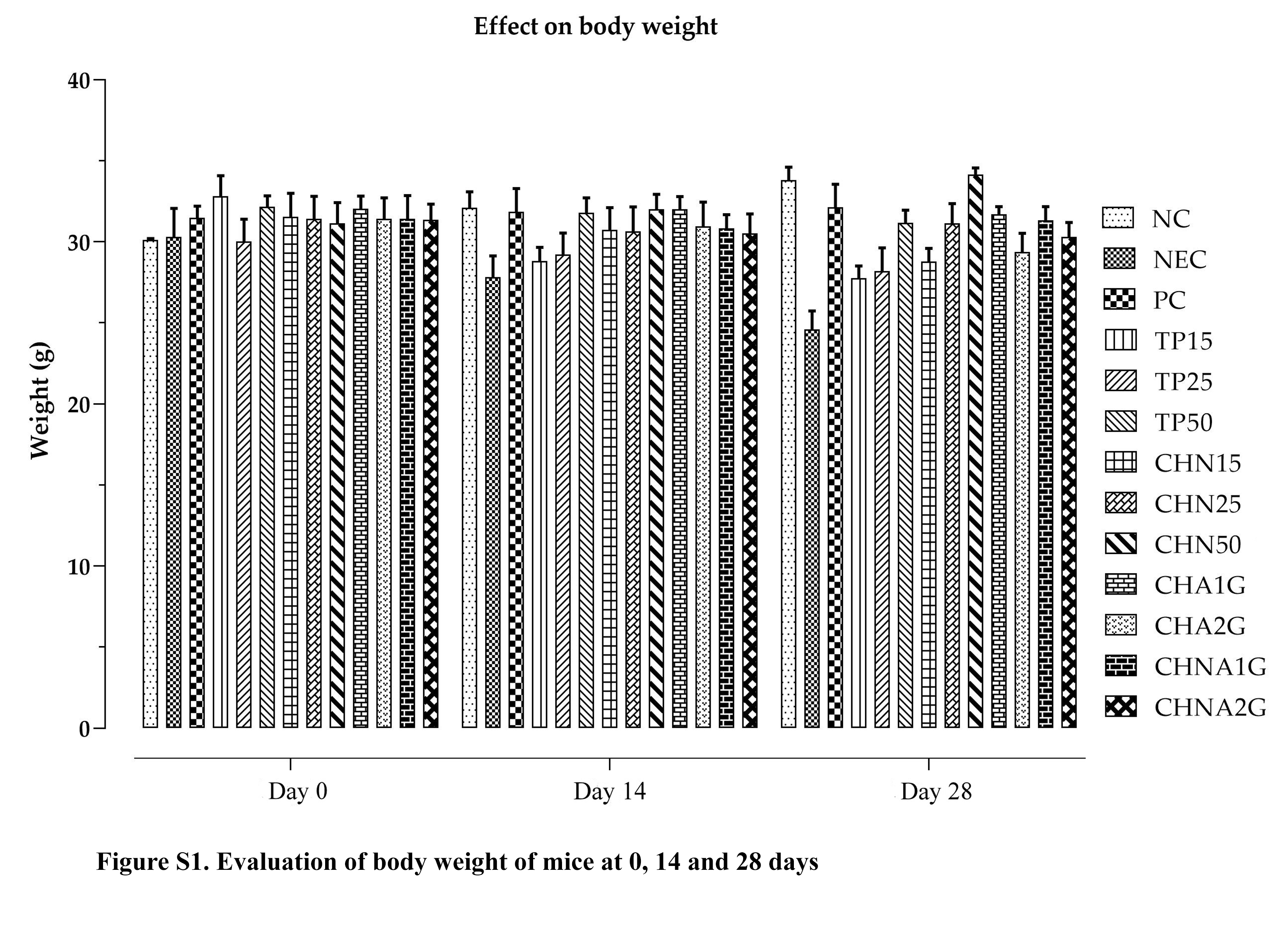

Supplement: Supplementary file 1 — Figure S1 [file FSB2-37-e22818-s002.tif]
