# Supplementary material for: Assessment of the antidiabetic potential of extract and novel phytoniosomes formulation of Tradescantia pallida leaves in the alloxan‐induced diabetic mouse model
Source: FASEB J. 2023 Mar 1;37(4):e22818. doi: 10.1096/fj.202201395RR (PMC11977607; doi:10.1096/fj.202201395RR)
Supplement: Supplementary file 2 — Appendix S1 [file FSB2-37-e22818-s001.docx]

**Table S1.** Evaluation of relative organ body weight in percentage

| **Groups** | **Liver** | **Pancreas** | **Kidney** |
| --- | --- | --- | --- |
| **NC** | 4.41 ± 0.80 | 5.58 ± 0.29 | 1.03 ± 0.07 |
| **NEC** | 6.50 ± 0.59^†***^ | 6.27 ± 0.38 | 0.99 ± 0.09 |
| **PC** | 4.71 ± 0.32^‡***¶*^ | 5.05 ± 0.53^‡**^ | 1.00 ± 0.06 |
| **TP15** | 5.54 ± 0.28^†***‡*^ | 5.74 ± 1.05 | 1.13 ± 0.15 |
| **TP25** | 5.41 ± 0.40^†**‡***^ | 5.60 ± 0.16 | 1.15 ± 0.12 |
| **TP50** | 4.87 ± 0.21^‡***^ | 5.09 ± 0.39^‡**^ | 1.05 ± 0.07 |
| **CHN15** | 5.30 ± 0.13^†*‡***^ | 5.43 ± 0.20 | 1.11 ± 0.05 |
| **CHN25** | 4.86 ± 0.22^‡***^ | 5.13 ± 0.18^‡**^ | 1.06 ± 0.06 |
| **CHN50** | 4.40 ± 0.09^‡¶***E**G*^ | 5.50 ± 0.20 | 1.01 ± 0.02 |
| **CHA1G** | 4.78 ± 0.15^‡***^ | 5.30 ± 0.14^‡*^ | 1.00 ± 0.01 |
| **CHA2G** | 4.89 ± 0.32^‡***^ | 5.50 ± 0.29 | 1.05 ± 0.10 |
| **CHNA1G** | 4.86 ± 0.21^‡***^ | 5.17 ± 0.33^‡**^ | 1.02 ± 0.01 |
| **CHNA2G** | 4.98 ± 0.13^‡***^ | 5.39 ± 0.26 | 1.08 ± 0.07 |

Values are presented in percentage ± SD. ^†^compared with NC, ^‡^compared with NEC, ^§^compared with PC, ^¶^compared with TP15, ^E^comparedwith TP25, ^F^compared with TP50,^G^compared with CHN15,^H^compared with CHN25,*^x^*compared with baseline.^*^p<0.05,^**^p<0.01,^***^p<0.001.Where NC – Normal Control, PC – Positive Contol, TP15 – extract group 15 mg/kg, TP25 – extract group 25 mg/kg, TP50 – extract group 50 mg/kg, CHN15 – niosome group 15 mg/kg, CHN25 – niosome group 25 mg/kg, CHN50 – niosome group 50mg/kg, CHA1G – extract 1000 mg/kg, CHA2G – extract 2000 mg/kg, CHNA1G – niosome 1000 mg/kg, CHNA2G – niosome 2000 mg/kg.
